# Silica is unlikely to be soluble in upper crustal carbonatite melts

**DOI:** 10.1038/s41467-023-35840-6

**Published:** 2023-02-21

**Authors:** Michael Anenburg, Tibor Guzmics

**Affiliations:** 1 Research School of Earth Sciences, Australian National University, Canberra, ACT 2600 Australia; 2 Lithosphere Fluid Research Lab, Eötvös University, Budapest, 1117 Hungary

**Keywords:** Petrology, Volcanology

**arising from** Berndt, J. & Klemme, S. *Nature Communications* 10.1038/s41467-022-30500-7 (2022)

A consensus among all experimental studies is that the solubility of silica (SiO_2_) is low in upper crustal carbonatite melts of below 5 kbar and 1000 °C (mostly much less than 5 wt% SiO_2_)^1,2^, in agreement with most natural melt inclusion studies^3–6^. Recently, Berndt and Klemme (B&K hereafter) documented haüyne-hosted melt inclusions from the Laacher See volcano exhibiting carbonatite–silicate liquid immiscibility formed at 720–880 °C and 1–2 kbar, with measured carbonatite melt containing high SiO_2_ (~ 15 wt%), and moderately low Na_2_O and K_2_O (combined contents below 8 wt%)^7^. Their reported silica contents are exceptionally high, never before observed in natural melt inclusions, and never synthesised in experimental studies at upper crustal conditions. If correct, their results significantly increase the permissible silica range contained in natural carbonatite melts, but our results show that their reported composition cannot be liquid, rejecting the existence silica-rich carbonatite melts at these conditions.

To test whether a silica-rich carbonatite melt can exist, we attempted to synthesise a melt with the average composition of B&K’s carbonatite melts (all in wt%, SiO_2_: 15.5%, TiO_2_: 0.57%, Al_2_O_3_: 1.07%, Cr_2_O_3_: 0.10%, FeO^t^: 2.72%, MnO: 0.94%, MgO: 0.53%, CaO: 50.8%, P_2_O_5_: 0.16%, Na_2_O: 3.76%, K_2_O: 0.55%, SO_3_: 0.41%, Cl: 0.44%, F: 4.04%, H_2_O: 5.06%, CO_2_: 35.3%). The percentage sums to ~122% due to excess CO_2_ in our experiment, compared to CO_2_ contents measured by B&K. This is to ensure stability of carbonatite melt, because low CO_2_ partial pressures will require unrealistically high alkali contents for immiscibility^1^. Temperature and pressure were set to 880 °C and 2 kbar, B&K’s upper limit. The Laacher See rocks formed at an oxygen fugacity of ΔNNO + 1 to +2^8^, so we attempted to use a Re–ReO_2_ oxygen buffer (≅ΔNNO + 2 at run conditions) by placing several layers of powdered Re metal with a tiny sprinkling of ReO_2_^9^. The starting materials were packed into a Ag_50_Pd_50_ capsule and run over 66 hours inside a piston cylinder using a 5/8” pressure vessel and an NaCl–Pyrex–MgO assembly. The experiment was quenched to room temperature, the capsule contents were exposed using sandpaper, and the contents were dried and then impregnated and reimpregnated with epoxy resin. All polishing and sample preparation was conducted dry to preserve potential water-soluble materials.

The experiment resulted in a mineral assemblage dominated by calcite and cuspidine (Fig. 1a, b). Accessory phases are perovskite, haüyne, and spinel. ReO_2_ reacted to form perrhenate (ReO_4_^–^) which was sequestered in the haüyne anion site. No ReO_2_ was found among the products, so oxygen fugacity was not strictly buffered to Re–ReO_2_, but the coexistence of Re^0^ metal and Re^7+^ perrhenate indicates it was not far from it. The resulting microstructure strongly resembles solid-state sintering, full of porosity and fine grain sizes of several micrometres. Liquid-like menisci and other wetting textures are not present. Carbonatite melts overwhelmingly quench to dendritic intergrowths of carbonate minerals^1,10–12^, which are likewise not present in this experiment. These observations indicate that no wide spread melting has occurred during the experiment. Rare features resembling melt pools (Fig. 1c) contain nanoscale grains of KReO_4_ (Fig. 1d), together with other elements (Fig. 1e). Although it is challenging to determine whether the detected elements originate from the melt pools or the surrounding minerals, the relatively high Ca and C counts indicate that a substantial CaCO_3_ component is present in this melt. The 1 atm melting point of KReO_4_ is roughly 550 °C, so we expect that its melting point at 2 kbar is below 880 °C. Thus, we inadvertently introduced a low melting point flux into the experiment by employing the Re-ReO_2_ buffer, and even then, no widespread melting of the capsule contents occurred. This demonstrates that the carbonatite melt composition reported by B&K cannot be liquid in upper crustal conditions.

**Fig. 1:**
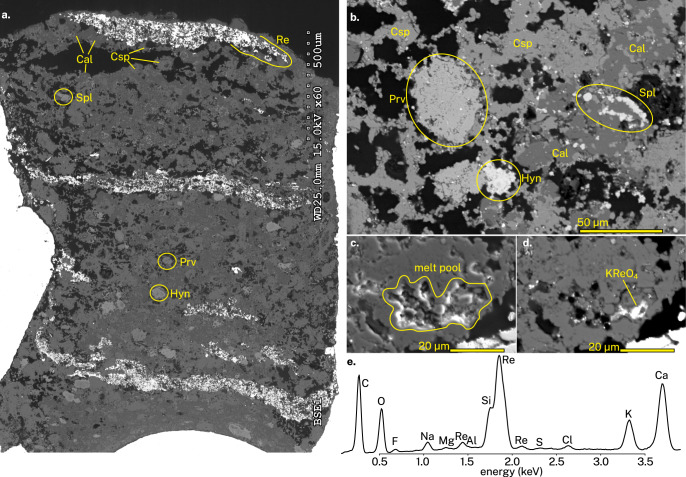
Experimental results. **a** backscattered electron image of the entire experimental capsule. Black regions are former CO_2_–H_2_O gas filled cavities, now impregnated with epoxy resin. Irregular white outline is the AgPd capsule. **b** closeup image of a representative region. **c** secondary electron image showing an area suspected as a former melt pool, enclosed by straight crystal faces. **d** backscattered electron image of the same region, showing bright potassium perrhenate. **e** an energy-dispersive X-ray spectroscopy (EDS) spectrum of the KReO_4_-rich area.

One may argue that the H_2_O/CO_2_ ratio in our experiment was not sufficiently high to trigger melting. This can be examined using thermodynamic relationships. The two dominant coexisting minerals are calcite and cuspidine, which can be linked by the reaction:

$${3{{{{{\rm{CaCO}}}}}}}_{3}+{{{{{{\rm{CaF}}}}}}}_{2}+{2{{{{{\rm{SiO}}}}}}}_{2}={{{{{{\rm{Ca}}}}}}}_{4}{{{{{{\rm{Si}}}}}}}_{2}{{{{{{\rm{O}}}}}}}_{7}{{{{{{\rm{F}}}}}}}_{2}+{3{{{{{\rm{CO}}}}}}}_{2}$$

Where CaF_2_ and SiO_2_ are thermodynamic components, and CO_2_ is the carbonic component of a mixed H_2_O–CO_2_ gas phase. Any increase in H_2_O would dilute the chemical potential of CO_2_, and given fixed F and Si contents (as constrained by B&K’s analytical results), would necessarily cause decomposition of calcite. Therefore, any H_2_O-induced melting of the composition would lead to a fluorinated hydrous calcsilicate melt, not a carbonatite. As for the effect of other experimental parameters, we cannot foresee a scenario in which moderate changes in either temperature, pressure, or composition can cause an essentially solid experiment to completely melt.

Fig. 2 shows compositions of immiscible silicate-carbonatite melts and silicate-saturated carbonatite melts at crustal pressures and temperatures between 650 and 1050 °C. It shows that natural and experimental carbonatite melts are on the right side of the solvus with low SiO_2_ (<4–5 wt%), regardless of their fluorine contents (up to 14 wt%). In contrast, B&K’s carbonatite melts are on the left side of the solvus, together with their presumed conjugate silicate melts^7^, and contain a silica/alkalis ratio too high for them to be immiscible between 650 and 1050 °C. These high-silica carbonatite melts may exist at mantle temperatures (>1200 °C) and pressures (>1.5 GPa), where the coexisting silicate melt cannot be an evolved phonolite with SiO_2_ contents higher than 57 wt% and MgO between 0.1 and 2.2 wt%^1,10,13,14^.

**Fig. 2:**
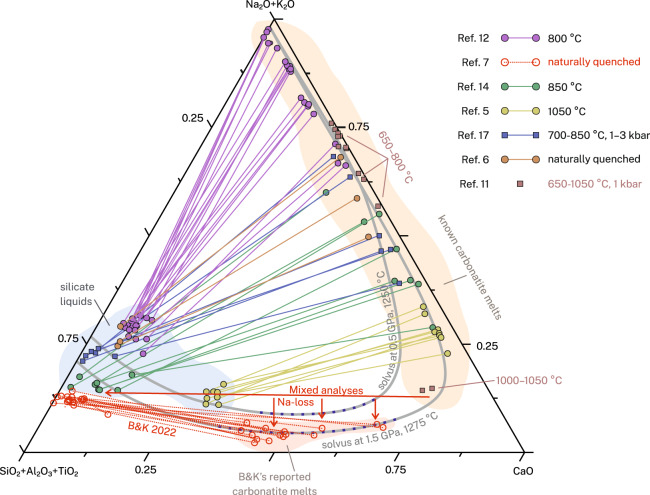
Composition of silicate-saturated carbonatite melts plotted on the pseudo-ternary diagram in the system Na_2_O+K_2_O−CaO-SiO_2_+Al_2_O_3_+TiO_2_−CO_2_ (all in wt%, projected from CO_2_). Squares and circles are experimental and melt inclusion data, respectively. Grey curves show experimental silicate-carbonatite solvi in the system Na_2_O−CaO−SiO_2_+Al_2_O_3_−CO_2_^1^. Purple dots on solvi show “single liquid” areas^1^. Solid and dotted tie lines connect the coexisting and immiscible silicate (left) and carbonatite (right) melt pairs. Temperatures next to brown squares indicate experimental temperature^11^. Carbonatite melt compositions with F > 3 wt% were corrected by assuming that all F is paired with Ca, with remaining Ca considered as CaO. Red arrows suggest possible analytical issues on the B&K carbonatite melt data.

We ascribe the spurious compositions of B&K’s reported carbonatite melts to three factors. First, alkali carbonates are extremely unstable under an electron beam. In a previous study we observed that Na counts drop substantially within just a few seconds, when measured using a 10 nA beam defocused to 15 μm^15^. B&K used a maximum spot size of 10 μm, in which the current density is 2.25 times higher. More commonly, they used smaller spot sizes (1–5 μm) in which current densities are at least an order of magnitude higher. We observed Na-loss on coarse grained crystalline materials^15^, whereas B&K’s are quenched liquids, consisting of nanoscale and potentially poorly crystalline phases. In such conditions, Na-loss is essentially instantaneous and even a short analysis time of 5 seconds strongly underestimates the Na contents of analysed materials (Fig. 2). Second, while B&K correctly state that their silicate melt compositions are contaminated by nanoscale droplets of carbonatite melt and correct for it, similar nanoscale droplets of silicate melt are observed in their carbonatite melts^7^. No correction was applied for these mixtures by B&K. Therefore, their reported carbonatite melt compositions are not pure and contain some degree of silicate melt contamination (Fig. 2). Finally, as our experiments resulted in haüyne, the same mineral host of B&K melt inclusions, this raises the possibility that some haüyne signal was measured together with the carbonatite melts.

To summarise, we could not replicate B&K’s findings in an experiment. Their results strongly deviate from possible carbonatite compositions based on previous phase equilibria and melt inclusion studies. Their analyses are likely to have suffered alkali loss during EPMA measurement. We therefore conclude that B&K do not provide evidence for silica-rich carbonatite melts in upper crustal conditions. Additionally, B&K’s findings cannot be a “missing link” between CaO-rich and extrusive Na-carbonatitic magmas^7^, as the link has already been described before^3,11,15–17^.

## Data Availability

All data and calculations require to produce Fig. 2 are available in the supplementary file. Source data are provided with this paper.

## Source data

Source Data
